# Histogram analysis of multiple mathematical diffusion-weighted imaging models for preoperative prediction of Ki-67 expression in hepatocellular carcinoma

**DOI:** 10.3389/fonc.2025.1531236

**Published:** 2025-03-11

**Authors:** Hongxiang Li, Jing Zhang, Baoer Liu, Zeyu Zheng, Yikai Xu

**Affiliations:** Department of Medical Imaging Center, Nanfang Hospital, Southern Medical University, Guangzhou, China

**Keywords:** hepatocellular carcinoma, diffusion, magnetic resonance imaging, multiple mathematical model, histogram analysis

## Abstract

**Objective:**

To explore whether a combination of clinico-radiological factors and histogram parameters based on monoexponential, biexponential, and stretched exponential models derived from the whole-tumor volume on diffusion-weighted imaging (DWI) could predict Ki-67 expression in hepatocellular carcinoma(HCC).

**Materials and Methods:**

Histogram parameters based on whole-tumor volumes were derived from monoexponential model, biexponential model, and stretched exponential model. Histogram parameters were compared between HCCs with high and low Ki-67 expression. Multivariate logistic regression and receiver operating characteristic curves were used to assess the ability to predict Ki-67 expression (expression index ≤ 20% vs. >20%).

**Results:**

In the training and test set, the 5th percentile of distributed diffusion coefficient (DDC) yielded the area under the curve (AUC) value of 0.816 (95% CI 0.713 to 0.894) and 0.867 (95% CI 0.655 to 0.972), respectively. Multivariable analysis showed that alpha-fetoprotein (AFP) level, skewness of perfusion fraction(f), and 5th percentile of DDC were independent predictors of high Ki-67 expression in HCCs. In the training and test sets, the AUC of the combined model for predicting high Ki-67 expression in HCCs were 0.902 (95% CI 0.814 to 0.957) and 0.908 (95% CI 0.707 to 0.989), respectively.

**Conclusion:**

Histogram parameters of multiple mathematical DWI models can be useful for predicting high Ki-67 expression in HCCs, and our combined model based on AFP level, skewness of f, and 5th percentile of DDC may be an effective approach for predicting Ki-67 expression in HCCs.

## 1 Introduction

Hepatocellular carcinoma (HCC) is the most common primary liver cancer and a major cause of cancer-related deaths worldwide (1). Despite substantial advancements in treatment strategies and imaging protocols, the prognosis of HCC remains poor, and recurrence rates after liver resection remain high (2, 3). Ki-67 is an important cell proliferation biomarker representing tumor aggressiveness. Previous studies have demonstrated that HCCs with high Ki-67 expression levels are associated with a significantly poor prognosis, including high recurrence rates and low recurrence-free survival and overall survival (4, 5). Moreover, some studies have shown that Ki-67-targeting therapy is an attractive and promising avenue in HCC treatment (6, 7). However, while current clinical practice guidelines recommend postoperative immunohistochemical examination as the gold standard for determining Ki-67 expression in HCCs, noninvasive preoperative evaluation of Ki-67 expression in HCCs may be beneficial for prognostic assessments and the development of personalized treatment strategies in clinical practice.

Preoperative imaging is a noninvasive and promising approach, but the existing detection methods for Ki-67 expression in HCC are challenging. Preliminary studies have shown that quantitative data, including apparent diffusion coefficient (ADC) values obtained from monoexponential model (MEM) and T1-mapping values can be used to evaluate Ki-67 expression in HCCs (8–10). Intravoxel incoherent motion parameters obtained from biexponential model (BEM) have also been used for evaluate Ki-67 expression in HCCs (11). Qualitative assessments, including evaluation of mosaic architecture, infiltrated appearance, and the targetoid hepatobiliary phase (HBP), have been reported to be useful for evaluating Ki-67 expression in HCCs before surgery (12). In addition, some studies have reported that deep learning and radiomics models are effective methods for predicting Ki-67 expression levels in HCCs (7, 13, 14).

Among advanced diffusion-weighted imaging (DWI) magnetic resonance imaging (MRI) models, the stretched exponential model (SEM) can provide more information related to diffusion and intravoxel heterogeneity in complex microstructures of biological tissue (15, 16). Since histogram analysis can reflect the subtle microscopic changes of pathology and tumor heterogeneity (17), our study aimed to explore the diagnostic performance of the combination of clinicoradiological factors with histogram parameters extracted from multiple mathematical DWI models (MEM, BEM, SEM) for noninvasive preoperative prediction of Ki-67 expression in HCCs.

## 2 Materials and methods

### 2.1 Study population

**Figure 1:**
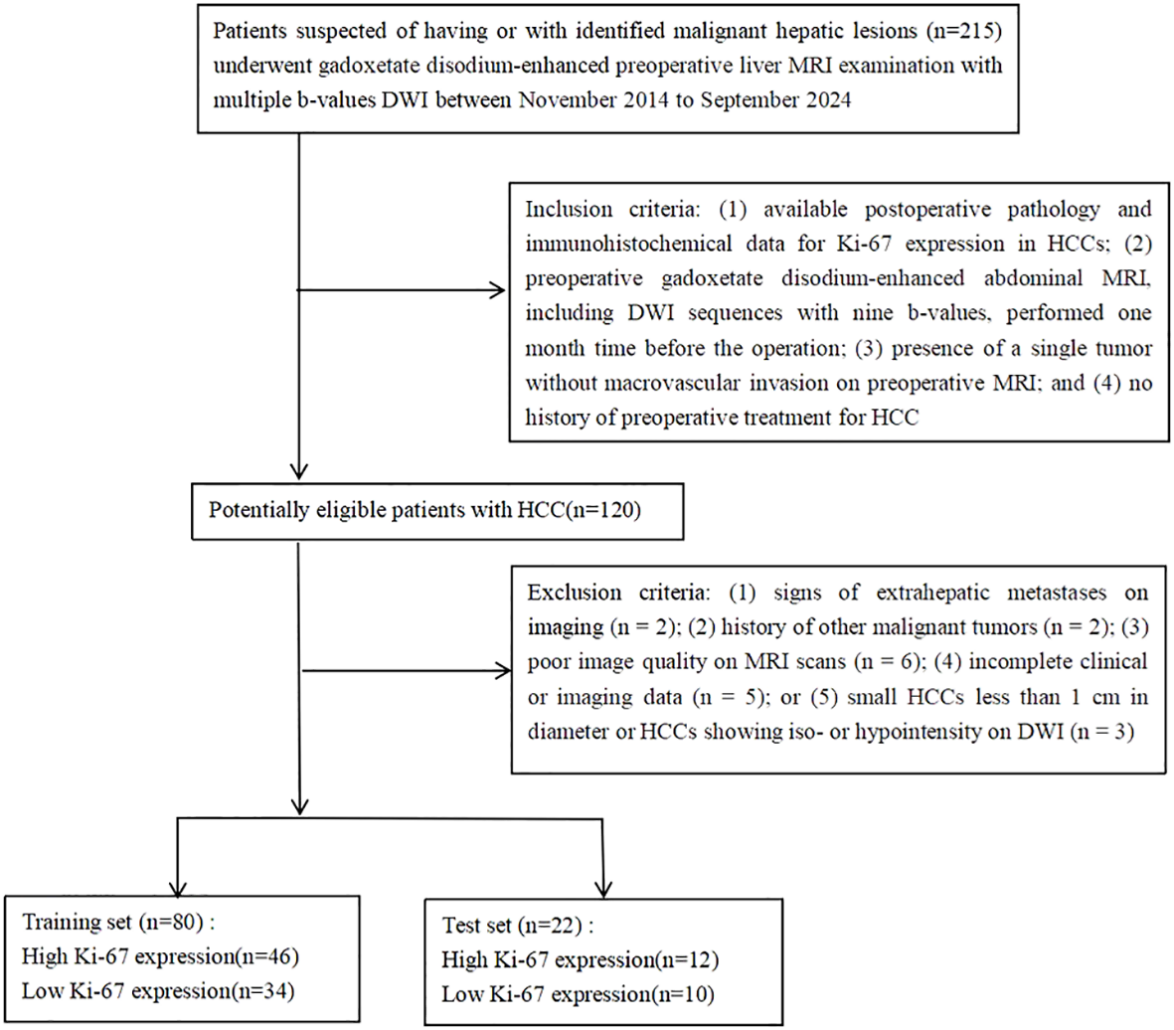
Patient enrollment flowchart.

This retrospective study was conducted in accordance with the Helsinki Declaration. Our Institutional Review Board approved this retrospective study (approval number: NFEC-202305-Y10), and all patients provided written informed consent. Between November 2014 to September 2024, 215 consecutive patients with suspected or confirmed malignant hepatic lesions underwent preoperative gadoxetate disodium-enhanced liver MRI examination with multiple b-values DWI sequence at our institution. The inclusion criteria were as follows: (1) available postoperative pathology and immunohistochemical data for Ki-67 expression in HCCs; (2) preoperative gadoxetate disodium-enhanced abdominal MRI, including DWI sequences with nine b-values, performed one month time before the operation; (3) presence of a single tumor without macrovascular invasion on preoperative MRI; and (4) no history of preoperative treatment for HCC such as radiofrequency ablation, transarterial chemoembolization, or other targeted therapies. The exclusion criteria were as follows: (1) signs of extrahepatic metastases on imaging (n = 2); (2) history of other malignant tumors (n = 2); (3) poor image quality on MRI scans (n = 6); (4) incomplete clinical or imaging data (n = 5); or (5) small HCCs less than 1 cm in diameter or HCCs showing iso- or hypointensity on DWI (n = 3). Finally, 102 patients who met the requirements of our study were included. HCC patients were randomly divided into a training set and a test set with a ratio of 8:2. Furthermore, 80 patients from the training set were used to establish the predictive model for Ki-67 expression. The predictive performance of the model was evaluated on the 22 cases from test set. The clinical information included data for age, sex, tumor size, alpha-fetoprotein (AFP) level, alanine transaminase (ALT), aspartate aminotransferase (AST), total bilirubin (TBIL), direct bilirubin (DBIL), indirect bilirubin (IBIL), albumin, platelet count, background liver parenchymal tissue, and origin of liver disease. The patient flowchart is shown in **Figure 1**.

### 2.2 MRI

All scans were performed on the Philips 3.0T MRI system (Achieva, Philips Healthcare, The Netherlands). All images were obtained by a 16-channel abdominal phased-array coil. Liver MRI was performed with the following sequences: (a) breath-hold, dual gradient-echo transverse T1-weighted in-phase and opposed-phase sequences; (b) transverse T2 -weighted spectral attenuated inversion recovery (SPAIR) sequences and T2-weighted sequences; (c) gadoxetate disodium-enhanced MRI using intravenous contrast agent (Primovist; Bayer Schering Pharma, Berlin, Germany; dose: 0.025 mmoL/kg) injection at a flow rate of 2.0 mL/s followed by a 20-mL saline flush and acquisition of arterial-phase, portal venous-phase, equilibrium-phase, and HBP images with a T1-weighted three-dimensional sequence with chemical selective fat-suppression sequences at 15–20 s, 40–60 s, 120–180 s, and 20 min; and (d) axial multiple b-value DWI pulse sequence obtained using respiratory-triggered single-shot echo planar imaging in the axial plane before gadoxetate disodium injection, and 9 b-values (b = 0, 10, 20, 40, 80, 200, 400, 600, and 1000 s/mm²). A more detailed description of the MRI sequences and parameters of MRI scans are shown in the **Supplementary Materials and methods 1**.

### 2.3 Histogram analysis

All histogram data were analyzed using in-house software based on 64-bit MATLAB 2014b (Math Works, Natick, MA) to acquire the histogram parameters of multiple mathematical DWI models and parametric maps. All nine b-values were used as input data. The histogram parameters of multiple mathematical DWI models were calculated according to the following mathematical models:

1. MEM parameters were calculated using the equation S(b)/S(0)=exp(–b × ADC), where S(b) is the mean signal intensity at the given b-value, and S0 is the mean signal intensity without diffusion gradient (18).
2. BEM parameters were calculated using the equation Sb/S0=(1-f)exp(−b×D)+fexp (−b×D*), where D* is the pseudo-diffusion coefficient linked to perfusion-related diffusion; D is the true diffusion coefficient that represents pure molecular diffusion; and f is the perfusion fraction related to microcirculation (19).
3. SEM parameters were acquired using the equation Sb/S0=exp[-(b × DDC)]α, where the distributed diffusion coefficient (DDC; the mean intravoxel diffusion rate) is the measure of signal decay rate with b, and α represents the water diffusion heterogeneity index, which reflects the intravoxel water molecular diffusion heterogeneity and ranges from 0 to 1 (15).

**Figure 2:**
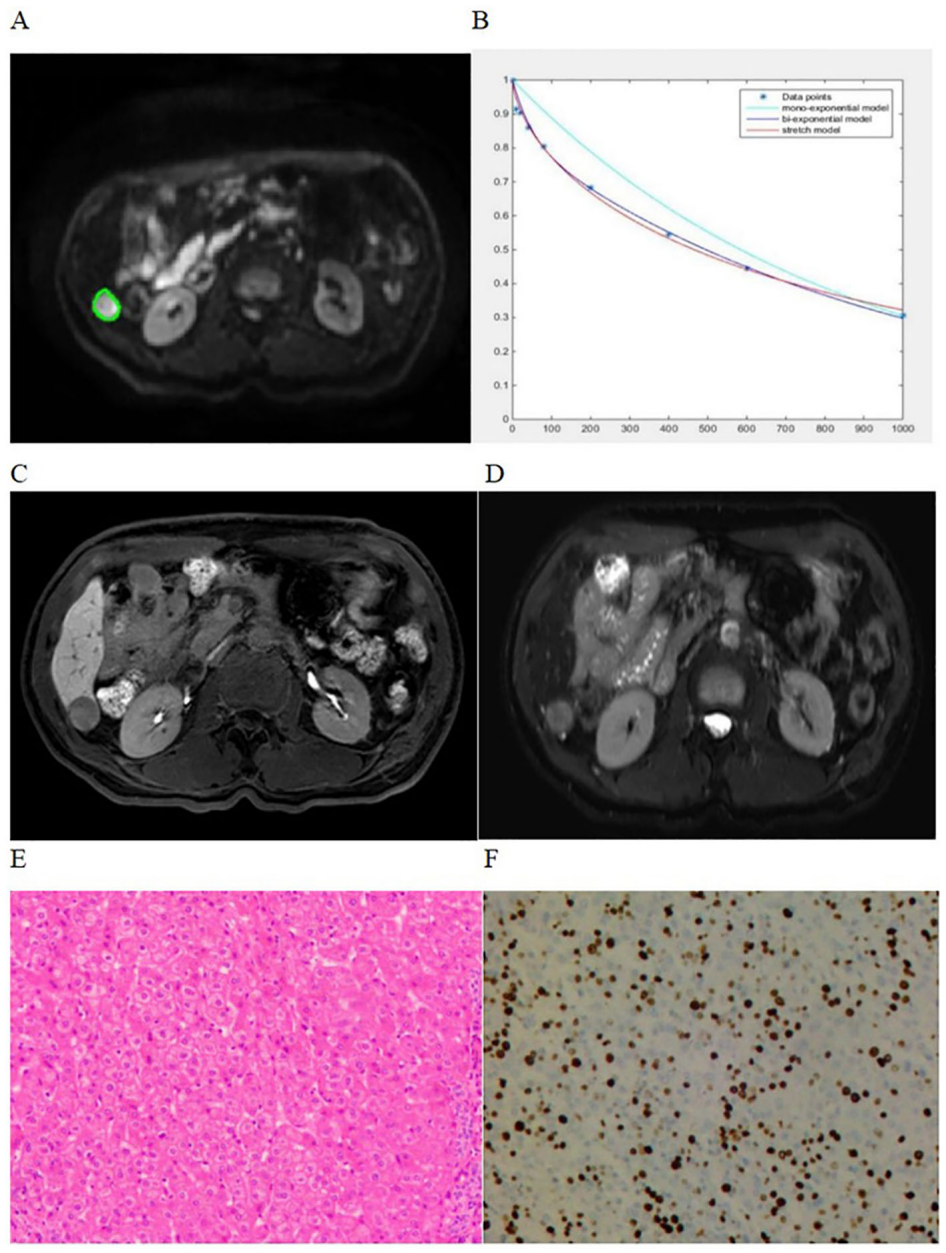
A 60-year-old man with high Ki-67 expression HCC showing how whole-tumor ROIs were delineated. Whole-tumor ROIs were derived by freehand drawing on **(A)** Each slice of tumor on the axial diffusion-weighted image (b =1000s/mm^2^). **(B)** Curves of different fits were derived from monoexponential, biexponential, and stretched exponential models. **(C, D)** Axial HBP and T2W-SPAIR images. **(E)** Pathological assessments showing HCC (hematoxylin-eosin, ×20). **(F)** Immunohistochemical assessments showing high proliferative activity of tumor cells with approximately 40% Ki-67 expression (×20).

For quantitative analysis, whole-tumor regions of interest (ROIs) were drawn independently by two radiologists with 6 and 10 years of experience in liver MRI who were blinded to the clinical and pathological findings. Each radiologist drew freehand ROIs around the tumor margin on axial DWI images of each tumor slice (b = 1000 s/mm^2^), and the ROIs were simultaneously copied to the ADC, D, D*, f, DDC, and α maps. The ROIs were delineated carefully to discard movement artifacts or image-degradation areas. Then, the ROIs were delineated to encompass as much of the entire lesion in each slice as possible. Axial T2-weighted images with SPAIR and HBP images were used as the reference to define the tumor border. A representative whole-tumor ROIs delineation of an HCC showing high Ki-67 expression is shown in **Figure 2**. All histogram parameters, including the mean, skewness, kurtosis, and percentile (5th, 50th, and 95th) values were derived from ADC, D, D*, f, DDC, and α maps for all mathematical DWI models. Calculation of all histogram metrics were performed on a per-voxel basis. The final values analysis were the average determined by two radiologists measurements.

### 2.4 Image analysis

All images were independently evaluated by two abdominal radiologists with 6 and 10 years of experience who were blinded to the clinical and pathological diagnosis. Interobserver agreement was assessed by Kappa (k) statistics, and any discrepancies were resolved by a third senior radiologist with over 20 years of experience in liver MRI. The two radiologists independently evaluated the following imaging features for each HCC: (a) non-rim arterial-phase hyperenhancement; (b) non-peripheral washout; (c) capsule appearance; (d) tumor hypointensity on HBP; (e) hemorrhage; (f) fat deposition; (g) corona enhancement; (h) peritumoral enhancement; and (i) the tumor margin. The detailed description of radiologic features are in the **Supplementary Materials and methods 2**.

### 2.5 Histopathological analysis

The pathological reports of all included patients with HCC were retrospectively reviewed. All pathologic examinations were performed by two pathologists with more than 10 years’ experience in liver pathology who were blinded to the radiological and clinical findings. Immunohistochemical staining for Ki-67 was performed, and the Ki-67 proliferation index was determined by measuring the frequency of cells staining positive for Ki-67. HCCs with ≤20% positively stained cells were classified as showing low Ki-67 expression, while those with >20% Ki-67-positive cells were classified as showing high Ki-67 expression in accordance with the approach described previously (13, 20).

### 2.6 Statistical analysis

Statistical analyses were performed using SPSS (version 20.0 for Windows, IBM Corporation, USA), Medcalc (version 18.2, Mariakerke, Belgium), and R software (version 4.4.1, http://www.r-project.org). The interobserver variabilities for histogram parameters were analyzed by calculating the interclass correlation coefficient (ICC). ICC value of >0.75 was considered to represent good agreement. Kappa (k) statistics were used to evaluate the agreement for radiological features (value of >0.75 was considered to represent good agreement). Continuous parameters were evaluated by independent t-test or Mann–Whitney U-test (non-normal distribution). Categorical variables were compared by the χ^2^ test or Fisher’s exact test. Univariate predictors with P < 0.05 were used in the multivariate logistic regression analysis to identify the independent risk factors for predicting Ki-67 expression. Collinearity analysis was also performed, and the Variance Inflation Factor (VIF) value>5 was considered to indicate collinearity between two variables. Receiver operating characteristic (ROC) curves were used to evaluate the diagnostic performance for preoperative identification of Ki-67 expression. Model fitting was assessed via calibration curves and the Hosmer-Lemeshow test. The clinical utility of the model was evaluated using decision curve analysis (DCA) by quantifying the net benefit under different threshold probabilities. Two-sided P values < 0.05 were considered to indicate statistical significance.

## 3 Results

### 3.1 Clinical characteristics

The baseline clinical characteristics of the training and test sets were similar (**Table 1**). A total of 80 patients from training set were enrolled in this study. Among these patients, 46 showed high Ki-67 expression (>20%) in the histopathological assessments, while the remaining 34 showed low Ki-67 expression (≤20%). Furthermore, 22 patients were included from test set, of which 10 had low Ki-67 expression (≤20%) and 12 had high Ki-67 expression (>20%). In the training set, the baseline clinical characteristics of HCC patients with high and low Ki-67 expression are shown in **Table 2**, And AFP level was significantly different between HCCs with high and low Ki-67 expression (P < 0.05).

**Table 1:** Baseline clinical characteristics of the training and test sets.

Variables	Training set (n = 80)	Test set (n =22)	*P* value
Age (years)	52.59 ± 11.73	56.36 ± 8.83	0.164^a^
Sex (male vs. female) (n)	70/10	21/1	0.498^C^
Tumor size, (cm)	2.25 ± 2.10	2.10 ± 3.58	0.867^b^
AFP level (n) (<20 ng/ml VS. ≥20ng/ml)	50/30	10/12	0.150^C^
ALT (IU/L)	27.50 ± 20.75	30.00 ± 24.25	0.339^b^
AST (IU/L)	26.00 ± 13.00	29.00 ± 16.00	0.211^b^
Total bilirubin (umol/L)	11.55 ± 7.48	13.50 ± 4.08	0.189^b^
Direct bilirubin (umol/L)	4.65 ± 3.38	4.40 ± 2.95	0.964^b^
Indirect bilirubin (umol/L)	7.10 ± 4.95	8.60 ± 3.68	0.159^b^
Albumin (g/L)	40.10 ± 7.55	39.40 ± 6.23	0.095^b^
Platelet count (×10^9^/L)	156.86 ± 54.89	132.63 ± 54.94	0.070^a^
Origin of liver disease (HBV vs. Other), (n)	76/4	22/0	0.575^C^
Background liver (cirrhosis or chronic hepatitis vs. non-cirrhotic chronic hepatitis) (n)	79/1	22/0	1.000^C^

AFP, alpha-fetoprotein; ALT, alanine aminotransferase; AST, aspartate aminotransferase; Data are presented as the mean ± standard deviation (normalized distribution) or median ± interquartile range (skewness distribution); Numbers, (n); ^a^Comparisons were performed by independent samples t-test;^b^Comparisons were performed by Mann−Whitney U test; ^C^Comparisons were performed by χ2 test; Significant results are in bold.

**Table 2:** Comparison of baseline clinical characteristics between HCCs with high and low Ki-67 expression in the training set.

Variables	High Ki-67 (n = 46)	Low Ki-67 (n =34)	*P* value
Age (years)	50.54 ± 11.49	55.35 ± 11.64	0.070^a^
Sex (male vs. female) (n)	40/6	30/4	1.000^C^
Tumor size, (cm)	2.15 ± 1.90	2.3 ± 1.90	0.495^b^
AFP level (n) (<20 ng/ml VS. ≥20ng/ml)	22/24	28/6	**0.002** ^C^
ALT (IU/L)	29.50 ± 22.50	26.00 ± 14.50	0.132^b^
AST (IU/L)	28.00 ± 13.75	24.00 ± 11.00	0.355^b^
Total bilirubin (umol/L)	11.15 ± 7.38	11.95 ± 8.35	0.480^b^
Direct bilirubin (umol/L)	4.30 ± 3.13	4.90 ± 3.43	0.422^b^
Indirect bilirubin (umol/L)	6.70 ± 4.83	7.30 ± 5.30	0.559^b^
Albumin (g/L)	39.65 ± 5.97	41.35 ± 6.90	0.216^b^
Platelet count (×10^9^/L)	152.41 ± 53.79	162.88 ± 56.59	0.403^a^
Origin of liver disease (HBV vs. Other), (n)	46/0	30/4	0.062^C^
Background liver (cirrhosis or chronic hepatitis vs. non-cirrhotic chronic hepatitis) (n)	46/0	33/1	0.425^C^

AFP, alpha-fetoprotein; ALT, alanine aminotransferase; AST,aspartate aminotransferase; Data are presented as the mean ± standard deviation (normalized distribution) or median ± interquartile range (skewness distribution); Numbers, (n); ^a^Comparisons were performed by independent samples t-test;^b^Comparisons were performed by Mann−Whitney U test; ^C^Comparisons were performed by χ2 test; Significant results are in bold.

### 3.2 Interobserver agreement in the training set

The ICC values indicated good-to-excellent agreement between the two abdominal radiologists in evaluating the histogram parameters of multiple mathematical DWI models (ICC = 0.75–0.97). The interobserver agreement between the two radiologists for evaluating radiologic features were good-to-excellent (k = 0.83-1.00). A detailed description of interobserver agreement are shown in **Supplementary Tables 4.1-4.3**.

### 3.3 Radiologic features of HCCs related to Ki-67 expression in the training set

Non-rim arterial-phase hyperenhancement, non-peripheral washout, capsule appearance, tumor margin, corona enhancement, peritumoral enhancement, fat deposition, hemorrhage, and tumor hypointensity on HBP did not statistically significant differences between HCCs with high and low Ki-67 expression in the training set (all P > 0.05, **Table 3**).

**Table 3:** Radiologic features between high and low Ki-67 expression HCCs in the training set.

Radiologic features	High Ki-67 (n=46)	Low Ki-67 (n=34)	*P* value
Non-rim arterial phase hyperenhancement	10	6	0.651
(Absent vs. Present)	36	28	
Non-peripheral washout	7	4	0.909
(Absent vs. Present)	39	30	
Capsule appearance	30	17	0.172
(Absent vs. Present)	16	17	
Tumor margin	26	17	0.563
(Non-smooth vs. Smooth)	20	17	
Corona enhancement	39	32	0.343
(Absent vs. Present)	7	2	
Peritumoral enhancement	43	31	1.000
(Absent vs. Present)	3	3	
Fat deposition	38	23	0.120
(Absent vs. Present)	8	11	
Hemorrhage	45	30	0.199
(Absent vs. Present)	1	4	
Tumor hypointensity on HBP	2	3	0.726
(Absent vs. Present)	44	31	

Data are numbers of lesions (n); HBP, hepatobiliary phase; Significant results are in bold.

### 3.4 Comparison of histogram parameters between HCCs with high and low Ki-67 expression

In the training set, the results of the comparison of all histogram parameters between HCCs with high and low Ki-67 expression are presented in **Tables 4.1** and **4.2**. HCCs with high Ki-67 expression had significantly lower mean and 5th and 50th percentile values for ADC than HCCs with low Ki-67 expression (all P < 0.05). HCCs with high Ki-67 expression showed significantly lower mean and 5th, 50th, and 95th percentile values for DDC than HCCs with low Ki-67 expression (all P < 0.05). HCCs with high Ki-67 expression showed significantly lower mean and 5th and 50th percentile values for D than HCCs with low Ki-67 expression (all P < 0.05). HCCs with high Ki-67 expression showed significantly lower mean and 50th percentile of f values than HCCs with low Ki-67 expression (all P < 0.05). Moreover, statistically significant differences in the skewness of ADC and f were observed between the two groups (all P < 0.05). However, the histogram parameters of α and D* showed no significant differences between HCCs with high and low Ki-67 expression (all P > 0.05). The distributions of histogram parameters based on multiple mathematical DWI models between a high Ki-67 expression HCC and a low Ki-67 expression HCC are shown in **Supplementary Figure S1**.

**Table 4.1:** Differences in histogram analyses of SEM and MEM between high and low Ki-67 expression HCCs in the training set.

Parameters	DDC (10^-3^ mm²/s)		P-value	α		P-value	ADC (10^-3^ mm²/s)		P-value
	High Ki-67	Low Ki-67		High Ki-67	Low Ki-67		High Ki-67	Low Ki-67	
Mean	0.96 ± 0.32	1.15 ± 0.60	**P<0.001** ^b^	0.65 ± 0.13	0.62 ± 0.13	P=0.271^a^	1.30 ± 0.32	1.50 ± 0.67	**P<0.001** ^b^
5th percentile	0.43 ± 0.25	0.65 ± 0.42	**P<0.001^b^**	0.31 ± 0.12	0.29 ± 0.14	P=0.567^a^	0.60 ± 0.23	0.91 ± 0.48	**P=0.001** ^b^
50th percentile	0.96 ± 0.33	1.10 ± 0.75	**P<0.001** ^b^	0.65 ± 0.17	0.62 ± 0.18	P=0.476^a^	1.20 ± 0.30	1.40 ± 0.70	**P<0.001** ^b^
95th percentile	1.65 ± 0.62	1.90 ± 0.45	**P=0.048** ^b^	0.97 ± 0.08	0.96 ± 0.09	P=0.551^a^	2.40 ± 0.87	2.79 ± 0.87	P=0.054^a^
kurtosis	3.36 ± 1.63	3.58 ± 3.49	P=0.425^b^	2.33 ± 1.10	2.30 ± 1.16	P=0.599^b^	5.66 ± 5.60	3.48 ± 5.76	P=0.058^b^
skewness	0.48 ± 0.75	0.17 ± 1.14	P=0.100^b^	0.01 ± 0.72	0.13 ± 0.54	P=0.383^a^	1.29 ± 1.13	0.68 ± 1.07	**P=0.019** ^b^

Data are presented as the mean±standard deviation (normalized distribution) or median±interquartile range (skewness distribution) ; ^a^Comparisons were performed by independent samples t-test; ^b^Comparisons were performed by Mann‒Whitney U test; Significant results are in bold.

**Table 4.2:** Differences in histogram parameters of BEM between high and low Ki-67 expression HCCs in the training set.

Parameters	D (10^-3^ mm²/s)		P-value	f		P-value	D* (10^-3^ mm²/s)		P-value
	High Ki-67	Low Ki-67		High Ki-67	Low Ki-67		High Ki-67	Low Ki-67	
Mean	0.83 ± 0.21	0.92 ± 0.47	**P=0.025** ^b^	0.13 ± 0.07	0.20 ± 0.13	P=**0.002** ^b^	59.48 ± 27.74	55.12 ± 32.96	P=0.523^a^
5th percentile	0.39 ± 0.21	0.61 ± 0.25	**P<0.001** ^a^	0.01 ± 0.03	0.02 ± 0.05	P=0.071^a^	3.00 ± 0	3.00 ± 0.00	P=0.340^b^
50th percentile	0.85 ± 0.21	0.92 ± 0.45	**P=0.027** ^b^	0.11 ± 0.09	0.19 ± 0.15	P=**0.001** ^b^	20.00 ± 26.00	21.00 ± 35.50	P=0.619^b^
95th percentile	1.20 ± 0.50	1.20 ± 0.52	P=0.406^b^	0.38 ± 0.11	0.44 ± 0.13	P=0.052^a^	200.00 ± 0.00	200.00 ± 87.00	P=0.073^b^
kurtosis	3.29 ± 1.88	3.55 ± 2.54	P=0.261^b^	3.37 ± 1.21	2.92 ± 1.09	P=0.087^a^	2.91 ± 4.11	3.65 ± 8.59	P=0.345^b^
skewness	-0.04 ± 1.07	-0.03 ± 1.26	P=0.566^b^	0.67 ± 0.60	0.27 ± 0.67	P=**0.001^b^**	1.12 ± 1.24	1.42 ± 2.08	P=0.572^b^

Data are presented as the mean ± standard deviation (normalized distribution) or median ± interquartile range (skewness distribution); ^a^Comparisons were performed by independent samples t-test;^b^Comparisons were performed by Mann−Whitney U test. Significant results are in bold.

In the test set, comparison of histogram parameters between HCCs with high and low Ki-67 expression are shown in **Supplementary Tables 1.1 and 1.2**.

### 3.5 Further diagnostic performance analysis in the training set

The results of ROC analyses of the significant histogram parameters in the training set are detailed in **Supplementary Table 2**. Among the histogram parameters of the training set, the 5th percentile of DDC yielded the highest AUC of 0.816 (95% CI 0.713, 0.894). The optimal cutoff value of the 5th percentile of DDC was 0.501× 10^−3^ mm²/s, with sensitivity and specificity of 73.9% and 76.5%, respectively.

### 3.6 Development and validation of predictive models for Ki-67 expression

**Figure 3:**
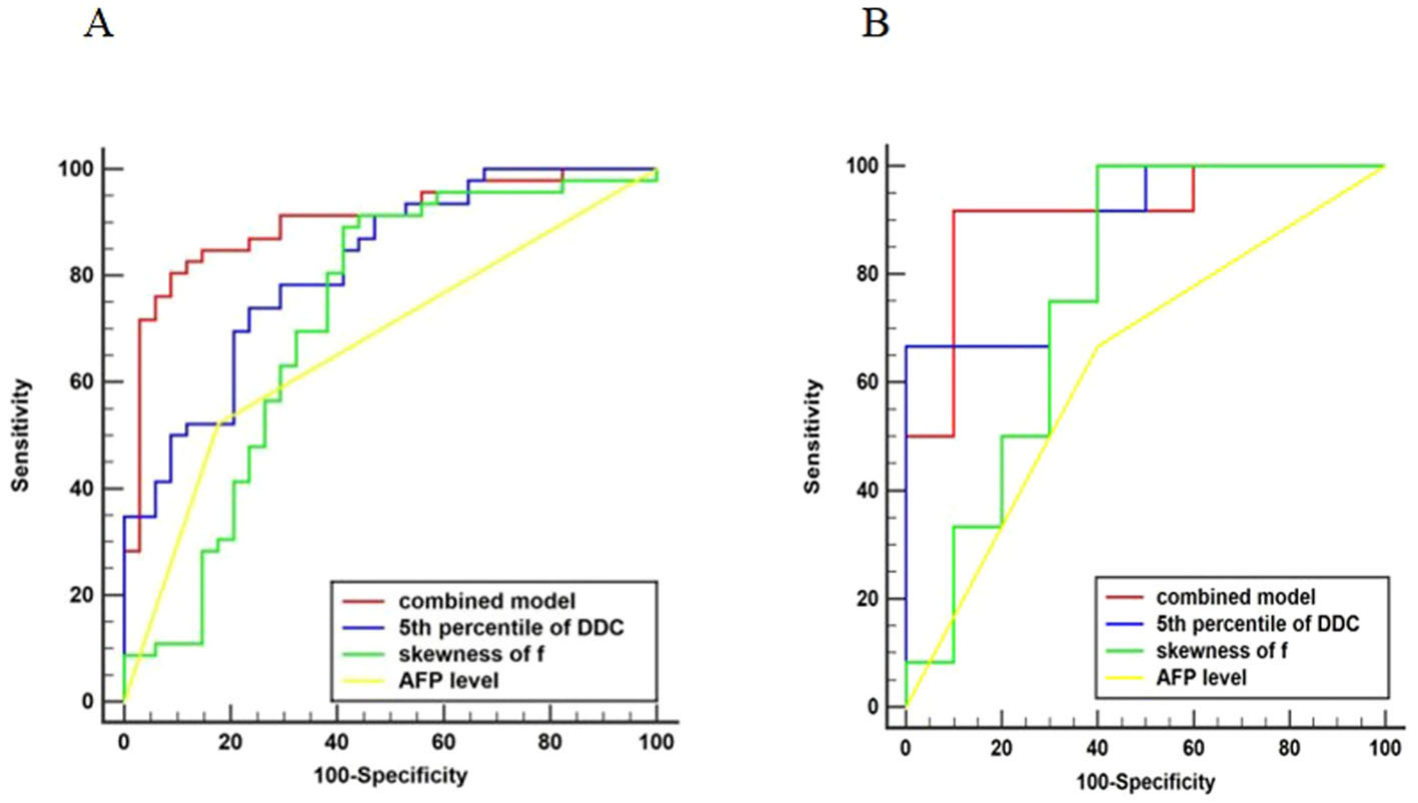
Comparison of ROC curves. Graph showing receiver operating characteristic curves for combined model, 5th percentile of DDC, skewness of f, and AFP level for identifying high Ki-67 expression in HCC. The AUC was the largest for the combined model in the training **(A)** and test **(B)** sets (AUC = 0.902 and 0.908, respectively).

Univariate analysis in the training set suggested that AFP level, histogram parameters(mean, 5th, 50th, and 95th percentiles) from DDC, histogram parameters (mean, 5th and 50th percentiles)from D, histogram parameters(skewness, mean, 5th, and 50th percentiles) from ADC, and histogram parameters (skewness, mean and 50th percentiles) from f were statistically significant variables (all P<0.05), and above-mentioned significant variables (P<0.05) were entered into the multivariate logistic regression using the forward method to identify the independent risk factors for Ki-67 expression. Collinearity analysis was performed for variables with P<0.05 after univariate analysis, and the VIF range was 1.050–2.882, indicating no collinearity between the variables. And both P values of Hosmer-Lemeshow test in the training (P=0.078) and test sets (P=0.112) were greater than 0.05, which showed that fitting of combined model was acceptable, it means no significant difference between the predicted probability and the actual probability. In the training set, multivariable logistic regression analysis showed that AFP level (P = 0.019, OR = 4.702), skewness of f (P = 0.049, OR = 3.364), and 5th percentile of DDC (P = 0.001, OR = 0.002) were independent predictors of HCCs with high Ki-67 expression (**Supplementary Table 3**). We constructed a combined model based on AFP level, skewness of f, and 5th percentile of DDC. The AUC of the combined model in the training and test sets were 0.902 (95% CI 0.814 to 0.957) and 0.908 (95% CI 0.707 to 0.989), respectively. Corresponding AUCs (95% CI), sensitivity, and specificity of the independent predictors and combined model are detailed in **Table 5**. **Figure 3** shows the comparisons of the ROC curves in the training and test sets. The DCAs for the combined model in the training set and test set are shown in **Supplementary Figure S2**. The combined model demonstrated good clinical utility by DCA analysis in both the training and test sets. Calibration curves of both the training and test sets are presented in **Supplementary Figure S3**, which showed predicted and actual probabilities of HCC with high Ki-67 expression.

**Table 5:** Diagnostic performance of the independent predictors and combined model.

Variables	Training set			Test set		
	AUC (95% CI)	Sensitivity (%)	Specificity (%)	AUC (95% CI)	Sensitivity (%)	Specificity (%)
AFP level	0.673 (95% CI 0.559, 0.773)	52.2	82.4	0.633 (95% CI 0.404, 0.826)	66.7	60.0
skewness of f	0.717 (95% CI 0.605, 0.812)	89.1	58.8	0.767 (95% CI 0.540, 0.918)	100	60.0
5th percentile of DDC	0.816 (95% CI 0.713, 0.894)	73.9	76.5	0.867 (95% CI 0.655, 0.972)	66.7	100
combined model	0.902 (95% CI 0.814, 0.957)	80.4	91.2	0.908 (95% CI 0.707, 0.989)	91.7	90.0

## 4 Discussion

In this study, we performed histogram analyses of multiple mathematical DWI models (MEM, BEM, SEM) to evaluate the Ki-67 expression (Ki-67 index ≤20% vs. >20%) in HCC. Our results showed that the histogram parameters of multiple mathematical DWI models can be used to quantify the Ki-67 expression of HCC. Moreover, we successfully developed a combined model based on AFP level, skewness of f, and 5th percentile of DDC. In the training and test sets, the combined model showed the best discriminative performance for predicting high and low Ki-67 expression in HCCs with an AUC of 0.902 and 0.908, respectively. This suggests that combining clinical information and histogram parameters can provide complementary information and improve predictive performance. Thus, this combined model demonstrated good predictive efficiency approach and clinical utility for preoperative prediction of high Ki-67 expression in patients with HCC. Our study have employed multivariate forward logistic regression analysis and model simplification techniques to avoid overfitting and improve predictive performance (21).

Many studies have confirmed that high Ki-67 expression levels are associated with rapid tumor progression, tumor invasiveness, and a poor prognosis in HCC patients (10, 22). In our study, none of the radiologic features showed statistically significant differences between HCCs with high and low Ki-67 expression in the train set. Our study results are consistent with the findings reported by Liang (12). One possible explanation is that radiologic features may have overlapped between HCCs with high and low Ki-67 expression levels when we adopted 20% as the cutoff value for Ki-67 expression. Nevertheless, the results across the existing studies have been rather inconsistent owing to selection bias, and further confirmation based on multi-center and large-sample studies is required. Nevertheless, our results also showed that the AFP level can be useful for predicting high Ki-67 expression in HCCs, which was consistent with previous studies (7, 9, 13). A high AFP level has been suggested to be associated with more biologically aggressive and unfavorable behaviors in HCCs, and high AFP levels are more likely to be observed in highly proliferative HCCs (7, 23).

Our study showed that histogram analyses based on MEM, BEM, and SEM were useful for predicting high Ki-67 expression in HCCs. ADC can provide quantitative information related to water diffusion and capillary perfusion. In the present study, the histogram-derived mean and 5th and 50th percentile values of ADC were significantly lower in HCCs with high Ki-67 expression than in HCCs with low Ki-67 expression, consistent with a previous study (8). This finding can be explained by the fact that HCCs with high Ki-67 expression have lower ADC values that represent the increasing levels of cellular components, which reduce the extracellular space and microcirculation perfusion in the tumor.

The D value has been suggested to more accurately reflect the diffusion of water molecules without being affected by capillary perfusion, making it more suitable than ADC values for detection of cell density in biological tissue (19, 24). In our study, the mean and 5th and 50th percentile values of D were significantly lower in HCCs with high Ki-67 expression than in HCCs with low Ki-67 expression. High Ki-67 expression in HCCs results in tumor cell proliferation and elevated nuclear-to-cytoplasmic ratios. Thus, lower percentile values of D may better reflect the focal tumor area with higher cellularity, which are associated with HCCs showing high Ki-67 expression (25). Moreover, D* and f have been reported to allow quantitative evaluation of capillary perfusion in HCC. Our results showed that the mean and 50th percentile values of f were significantly lower in the HCCs with high Ki-67 expression compared to HCCs with low Ki-67 expression. HCC malignancy manifesting as an increase in the expression of Ki-67 is also characterized by abnormal vascular branching patterns of tumor capillaries, an incomplete basal membrane, and blood flow into small, leaky, and poorly efficient tumor capillaries, all of which increase the incidence of leaks and decrease capillary perfusion (11, 24). However, the histogram D* values showed no significant differences between HCCs with high and low Ki-67 expression. The unsatisfactory performance of D* may be associated with the poor reproducibility of this parameter, which shows substantial fluctuations and lacks stability (25–27). Interestingly, HCCs with high Ki-67 expression showed significantly higher skewness for f values than HCCs with low Ki-67 expression. Moreover, the skewness of f values was an independent predictor in the multivariable logistic regression analysis. Since skewness reflects the normality of the histogram distribution, this finding may indicate greater complexity of perfusion in HCCs with high Ki-67 expression (17). We speculated that changes in tissue perfusion skewness may be a relatively vital factor in the prediction of Ki-67 expression in HCCs.

The SEM can simultaneously provide information about the non-Gaussian behavior of molecular diffusion and heterogeneity (27, 28). The SEM may also reflect complex microstructures in biological tissue, and has the potential to provide more information about the non-Gaussian distribution caused by restricted diffusion of water molecules (28). The DDC, which reflects a continuous distribution of diffusion coefficients from each diffusion compartment, has been previously reported to be more accurate than conventional ADC or D values for liver fibrosis staging and assessment of focal liver lesions (26, 27). In our study, we found the 5th percentile of DDC was an independent predictor for evaluating HCCs with high Ki-67 expression in the multivariable logistic regression analysis compared to the histogram values of ADC and D. And the 5th percentile of DDC showed superior performance in evaluating HCCs with high Ki-67 expression. The HCCs with high Ki-67 expression showed faster cell proliferation than those with low Ki-67 expression, which resulted in increased nucleus/cytoplasm ratio and decreased extracellular/intracellular space. This histopathological characteristics can lead to increasing cell density and complicated tumor microenvironment. Thus, HCCs with high Ki-67 expression may show more complicated non-Gaussian diffusion behavior than Gaussian diffusion behavior in tumor microenvironment; and the DDC may theoretically provide a more accurate depiction of diffusion and yield more information on non-Gaussian distribution of tumor microenvironment. Moreover, the lower percentile values of DDC, especially the 5th percentile value, may better reflect focal areas of densely packed cell components in HCCs with high Ki-67 expression (16, 28, 29).

The α value has been reported to be associated with the deviation of water diffusion, which represents the tissue heterogeneity (30–32). However, no significant differences was observed in the histogram α values between HCCs showing high and low Ki-67 expression, likely because α may be insensitive to the differences in microenvironment between HCCs with high and low Ki-67 expression. Thus, the underlying biological basis for the use of α values in evaluating Ki-67 expression in HCCs remains unclear. Future studies should aim to investigate the usefulness of histogram α values in a larger sample size.

Our study had several limitations. First, our study was performed at a single institution with a relatively small population; therefore, conducting multi-center external validation, taking cross-validation, and expanding the dataset will be needed in our future research in order to ascertain the generalizability of the model on a larger scale and the model robustness. Second, while multiexponential diffusion attenuation could be more significant with higher b-values for HCC, higher b-values are linked to loss of SNR, which may affect the accuracy of calculated parameters. Thus, the highest b-value in our study was only 1000 owing to relatively longer examination time and limitations in the SNR (32). Third, the minimum and maximum values were highly sensitive to artifact and noise in the maps (29, 33), whereas the 5th and 95th percentile values were less affected by these factors. Therefore, analyses using the 5th and 95th percentile values may be more reliable for histogram analysis. Fourth, the calibration curves were not very ideal due to the relatively small sample size. However, the mean absolute error (MAE) values of the calibration curves in both the training and test sets were less than 0.1, which indicated that the overall predictive performance of the MAE in the training and test sets were acceptable. In general, the overall fitting effect of our combined model through MAE analysis and Hosmer-Lemeshow test is reasonable. Thus, there is room for improvement in the calibration curve, which needs to be further optimized through large-scale and multi-center studies. Finally, this study aimed to explore weather a combination model of clinico-radiological factors and histogram parameters could preoperatively predict Ki-67 expression in HCC. Future research, we will incorporate survival analysis to further prove the clinical applicability and significance of this model through artificial intelligence method (34).

In conclusion, our preliminary results suggest that multiple mathematical DWI models could be used to assess Ki-67 expression in HCCs. Furthermore, our combined model based on AFP level, skewness of f, and 5th percentile of DDC exhibited remarkable predictive power for preoperative prediction of Ki-67 expression (index ≤20% vs.>20%) in HCCs.

## Data Availability

The original contributions presented in the study are included in the article/**Supplementary Material**. Further inquiries can be directed to the corresponding author.
