# Supplementary material for: Histogram analysis of multiple mathematical diffusion-weighted imaging models for preoperative prediction of Ki-67 expression in hepatocellular carcinoma
Source: Front Oncol. 2025 Mar 11;15:1531236. doi: 10.3389/fonc.2025.1531236 (PMC11932891; doi:10.3389/fonc.2025.1531236)
Supplement: Supplementary file 1 [file DataSheet1.pdf]

Supplementary Materials

Supplementary materials and methods

supplementary materials and methods 1:MR scan sequence and parameters

Liver MRI was performed with the following sequences: (a) breath-hold, dual gradient-echo transverse T1-weighted in-phase and opposed-phase sequences with the following parameters: repetition time (TR) = 3.4 ms, echo time (TE) = 1.32 ms, matrix = 252 × 203, and slice thickness = 5 mm; (b) transverse T2 -weighted spectral attenuated inversion recovery (SPAIR) sequences with the following parameters: TR/TE = 615 ms/70 ms, matrix = 268 × 163, and slice thickness = 5 mm, and T2-weighted sequences with the following parameters: TR/TE = 651 ms/80 ms, matrix = 288 × 187, and slice thickness = 5 mm; (c) gadoxetate disodium-enhanced MRI using intravenous contrast agent (Primovist; Bayer Schering Pharma, Berlin, Germany; dose: 0.025 mmoL/kg) injection at a flow rate of 2.0 mL/s followed by a 20-mL saline flush and acquisition of arterial-phase, portal venous-phase, equilibrium-phase, and HBP images with a T1-weighted three-dimensional sequence with chemical selective fat-suppression sequences at 15–20 s, 40–60 s, 120–180 s, and 20 min using the following parameters: TR/TE = 3.2 ms/1.53 ms, matrix = 304 × 239, and slice thickness=5 mm; and (d) axial multiple b-value DWI pulse sequence obtained using respiratory-triggered single-shot echo planar imaging (echo planar imaging factor, 57) in the axial plane before gadoxetate disodium injection with the following parameters: TR/TE = 1967/57, matrix = 132 × 114, slice thickness = 5 mm, number of signals averaged = 2, slice gap = 0.5 mm, slices = 36, and 9 b-values (b = 0, 10, 20, 40, 80, 200,400, 600, and 1000 s/mm²).

supplementary materials and methods 2: Image analysis

The two radiologists independently evaluated the following imaging features for each HCC: (a) non-rim arterial-phase hyperenhancement, which was defined as tumor signal intensities unequivocally greater in whole or in part than liver in arterial phase; (b) non-peripheral washout, which was identified by a temporal reduction in the enhancement of the tumor in the whole liver or a part relative to composite liver tissue in the portal and equilibrium phases; (c) capsule appearance, which was characterized by a smooth, uniform, sharp enhancing rim border in the portal and equilibrium; (d) tumor hypointensity on HBP, which was determined by comparing the main signal intensities of the lesions with liver parenchyma and defining the tumor as hypo, iso, or hyperintense; (e) hemorrhage, which was defined as intralesional or perilesional hemorrhage in the absence of biopsy, trauma, or intervention; (f) fat deposition, which was assessed using T1-weighted in-phase and opposed-phase chemical-shift imaging; (g) corona enhancement, which was defined as periobservational enhancement in the late arterial phase or early portal phase; (h) peritumoral enhancement, which was defined as the detectable portion with arterial-phase enhancement located outside the tumor border that turned isointense with background liver parenchyma in the portal or equilibrium phase; and (i) the tumor margin, which was categorized as smooth (a smooth border on HBP images), or non-smooth (focal extranodular growth, confluent

multinodular growth, or infiltrative borders with irregular shape).

Supplementary tables

supplementary table1.1 Differences in histogram analyses of SEM and MEM between high and low Ki-67 expression HCCs in the test set

Parameters	DDC (10^{-3} mm²/s)		P-value	α		P-value	ADC (10^{-3} mm²/s)		P-value
	High Ki-67	Low Ki-67		High Ki-67	Low Ki-67		High Ki-67	Low Ki-67	
Mean	0.95±0.25	1.27±0.26	**P=0.009**[a]	0.72±0.12	0.65±0.11	P=0.194[a]	1.29±0.42	1.69±0.43	**P=0.046**[a]
5th percentile	0.32±0.21	0.69±0.20	**P<0.001**[a]	0.75±0.17	0.66±0.15	P=0.715[a]	0.43±0.27	0.84±0.28	**P=0.003**[a]
50th percentile	0.91±0.27	1.30±0.36	**P=0.009**[a]	0.31±0.16	0.29±0.08	P=0.255[a]	1.10±0.47	1.45±0.40	**P=0.036**[b]
95th percentile	1.90±0.62	2.00±0.30	P=0.300[b]	1.00±0.00	1.00±0.00	P=0.843[b]	2.60±0.99	3.03±0.88	P=0.303[a]
kurtosis	3.13±5.41	2.99±1.40	P=0.947[b]	2.15±1.72	2.52±0.81	P=0.356[b]	5.97±5.24	4.45±5.82	P=0.553[b]
skewness	0.34±0.89	0.31±1.04	P=0.429[b]	-0.57±0.61	-0.14±0.54	P=0.104[a]	1.49±0.87	1.22±0.72	P=0.444[a]

supplementary table1.2 Differences in histogram parameters of BEM between high and low Ki-67 expression HCCs in the test set

Parameters	D (10^{-3} mm²/s)		P-value	f		P-value	D* (10^{-3} mm²/s)		P-value
	High Ki-67	Low Ki-67		High Ki-67	Low Ki-67		High Ki-67	Low Ki-67	
Mean	0.81±0.18	1.05±0.27	**P=0.021**[a]	0.12±0.05	0.18±0.07	**P=0.042**[a]	50.25±26.35	55.60±19.03	P=0.598[a]
5th percentile	0.37±0.21	0.64±0.17	**P=0.004**[a]	0.01±0.03	0.01±0.01	P=0.361[a]	3.00±0.75	3.00±0.25	P=0.613[b]
50th percentile	0.79±0.19	1.08±0.28	**P=0.012**[a]	0.08±0.06	0.16±0.09	**P=0.047**[a]	12.00±40.75	25.00±28.00	P=0.467[b]
95th percentile	1.28±0.33	1.46±0.42	P=0.298[a]	0.37±0.19	0.41±0.10	P=0.602[a]	200±0.00	200±0.25	P=0.922[b]
kurtosis	3.17±1.61	3.80±1.72	P=0.391[a]	4.02±2.51	2.68±1.62	P=0.210[b]	3.43±5.11	3.06±1.71	P=0.429[b]
skewness	0.30±0.61	0.09±0.58	P=0.427[a]	0.98±0.79	0.37±0.85	**P=0.035**[b]	1.45±1.47	1.05±0.60	P=0.323[b]

Data are presented as the mean±standard deviation (normalized distribution) or median±interquartile range (skewness distribution) ; [a]Comparisons were performed by independent samples t-test; [b]Comparisons were performed by Mann–Whitney U test; Significant results are in bold.

supplementary table2. Diagnostic performance of significant histogram parameters for identifying HCCs with high Ki-67 expression in the training set.

Parameters	AUC	P Value	Cutoff value	Sensitivity(%)	Specificity(%)
5th percentile of ADC	0.728	＜0.001	≤0.653	65.2	76.5
mean of ADC	0.735	＜0.001	≤1.3	63.0	76.5
50th percentile of ADC	0.760	＜0.001	≤1.2	63.0	76.5
skewness of ADC	0.654	0.02	＞0.819	76.1	76.0
mean of f	0.707	0.001	≤0.211	89.1	50.0
50th percentile of f	0.715	＜0.001	≤0.125	58.7	79.4
skewness of f	0.717	＜0.001	＞0.34	89.1	58.8
5th percentile of D	0.749	＜0.001	≤0.558	82.6	58.8
mean of D	0.648	0.023	≤0.971	87.0	41.2
50th percentile of D	0.645	0.025	≤0.917	76.1	52.9
5th percentile of DDC	0.816	＜0.001	≤0.501	73.9	76.5
95th percentile of DDC	0.626	0.039	≤1.6	50.0	73.5
mean of DDC	0.764	＜0.001	≤0.915	45.7	94.1
50th percentile of DDC	0.742	＜0.001	≤0.89	41.3	97.1

Units of $\times 10^{-3} mm^2/s$ for histogram ADC, D, and DDC.

supplementary table3. Multivariate analysis with logistic regression for identifying HCCs with high Ki-67 expression in the training set.

Parameters	B	P Value	OR（95% CI）
skewness of f	1.213	0.049	3.364（1.004-11.270）
5th percentile of DDC	-6.328	0.001	0.002（0-0.066）
AFP level	1.548	0.019	4.702（1.294-17.083）

supplementary table4.1. ICC results for the histogram parameters of SEM and MEM in the training set.

DDC Histogram Parameter	ICC	α Histogram Parameter	ICC	ADC Histogram Parameter	ICC
Mean	0.89	Mean	0.95	Mean	0.85
5th percentile	0.93	5th percentile	0.81	5th percentile	0.93
50th percentile	0.95	50th percentile	0.89	50th percentile	0.92
95th percentile	0.75	95th percentile	0.87	95th percentile	0.78
kurtosis	0.77	kurtosis	0.75	kurtosis	0.76
skewness	0.75	skewness	0.91	skewness	0.76

supplementary table4.2. ICC results for the histogram parameters of BEM in the training set.

D Histogram Parameter	ICC	f Histogram Parameter	ICC	D*Histogram Parameter	ICC
Mean	0.90	Mean	0.94	Mean	0.94
5th percentile	0.75	5th percentile	0.97	5th percentile	0.75
50th percentile	0.95	50th percentile	0.97	50th percentile	0.96
95th percentile	0.84	95th percentile	0.82	95th percentile	0.89
kurtosis	0.77	kurtosis	0.76	kurtosis	0.87
skewness	0.79	skewness	0.86	skewness	0.86

supplementary table4.3. Kappa (k) statistics for the radiologic features in the tr aining set.

Radiologic features	Kappa (k) statistics
Non-rim arterial phase hyperenhancement	0.898
Non-peripheral washout	0.885
Capsule appearance	0.843
Tumor margin	0.846
Corona enhancement	0.866
Peritumoral enhancement	1.000
Fat deposition	0.885
Hemorrhage	0.835

Tumor hypointensity on HBP	0.835

supplementary figure s1

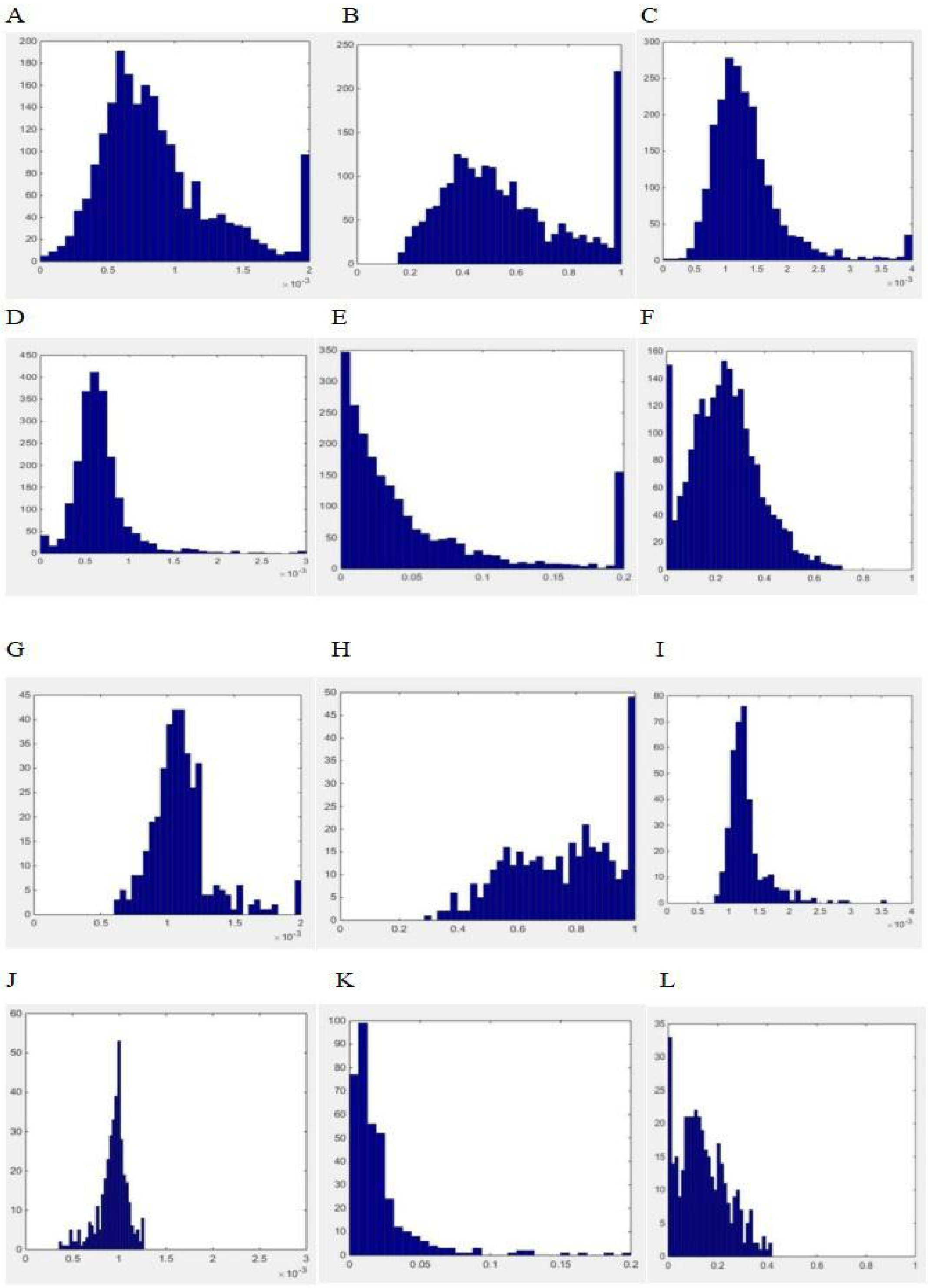

supplementary figures s1 caption

supplementary figure s1. Histogram distributions of multiparameters DWI between a high Ki-67 expression HCC and a low Ki-67 expression HCC. Histograms of DDC,α,ADC,D,D*,and f in a HCC with high Ki-67 expression (A, B, C, D, E, and F respectively). Histograms of DDC,α,ADC,D,D*,and f in a HCC with low Ki-67 expression (G, H, I, J, K, and L, respectively). The transverse axis represents the cumulative frequency distributions of the histogram parameters, while the vertical axis represents the pixel number count of each parameter.

supplementary figure s2

A

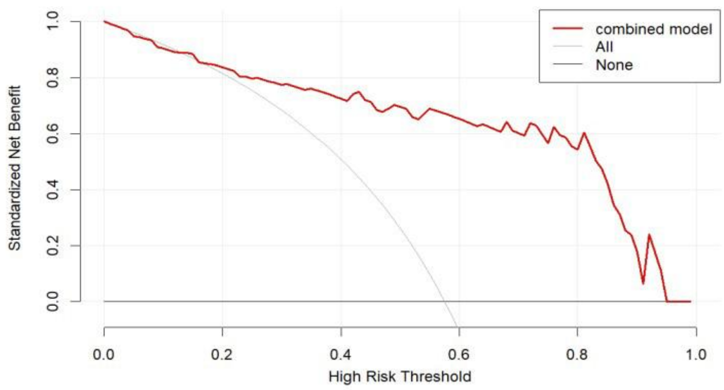

B

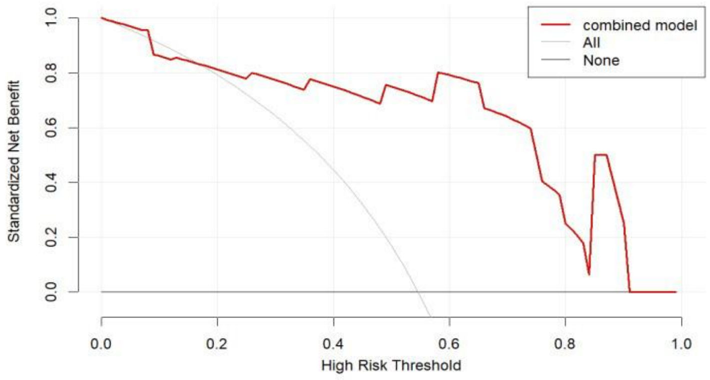

supplementary figures s2 caption

The DCAs for the combined models of the training set (A) and test set(B).

supplementary figures s3

A

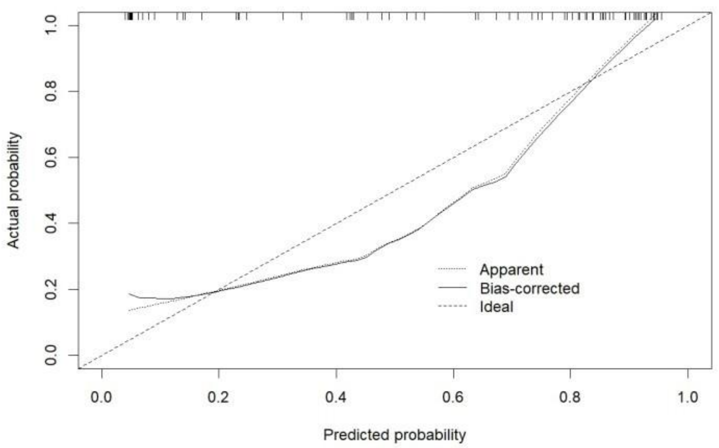

B

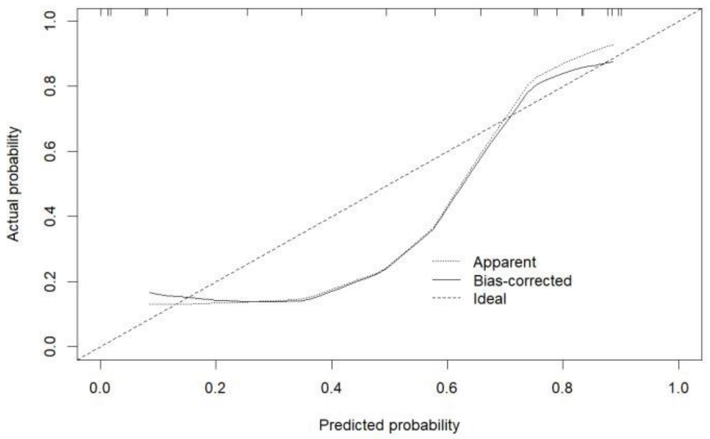

supplementary figures s3 caption

The calibration curves of the combined models for predicting Ki-67 expressioni n both the training set (A) and test set (B). The curves assess the model fittin g. The x-axis represents the predicted probability, and the y-axis represents the actual probability.
